# Supplementary material for: The life expectancy of older couples and surviving spouses
Source: PLoS One. 2021 May 14;16(5):e0250564. doi: 10.1371/journal.pone.0250564 (PMC8121294; doi:10.1371/journal.pone.0250564)
Supplement: Attachment — Submitted filename: Referee report PONE-D-20-17148.pdf [file pone.0250564.s001.pdf]

<u>Referee report PONE-D-20-17148</u>

Robert Pollak and Janice Compton

The Life Expectancy of Older Couples and Surviving Couples

Short summary

This paper analyzes the life expectancy of married couples across race/ethnicity and education groups. The main point of the paper is to document that joint life expectancies and life expectancies of surviving spouses are different when calculated based on Census and NCHS data rather than just based on individual life expectancies. This is due to a substantial overlap in spouses' mortality distributions. The paper also shows that differences in life expectancies by education are greater than the racial differences.

General comments

The paper makes a nice descriptive empirical contribution to the literature by documenting the importance of taking into account within-couple mortality correlations, which impact life expectancies of married couples. Although this point seems obvious, this is important knowledge for individual planning and for policy makers, and the authors conclude that tools such as the "Life Expectancy Calculator" on the Social Security website could be augmented to calculate point and survivor life expectancies.

Generally, the empirical analysis seems convincing, although it is not always quite clear what the authors have done in every step, and how the data was used.

My comments are mainly centered around the structure of the paper and the way that the analysis is documented.

1) I think the paper would be more interesting to read if it more clearly explained early on in the paper, perhaps in the Introduction, why we would expect that it is important to take within-couple mortality correlations into account. The background and previous literature on intra-marriage correlations and sorting into marriage is not introduced and discussed before 7 pages into the paper (lines 181-195), and the literature survey is very short. The paper would improve by extending the literature discussion on why we should expect intra-spouse correlation in health and mortality, citing papers on 1) assortative mating in health, wealth etc. (e.g. Guner, Kulikova & Llull in *European Economic Review*, Vol. 104, May 2018) and selection into marriage, 2) intra-marriage correlation in health behavior through habits, smoking, drinking, exercise, diet etc., as well as information exchange within couples (e.g. Fadlon and Nielsen, 2019, *American Economic Review*, "Family Health Behaviors"), 3) coordination of couples' retirement decisions that may impact health and life expectancy (e.g. Gustman and Steinmeier 2000 in *Journal of Labor Economics*). This section may also include a short discussion of health effects of bereavement of a spouse.

2) The structure of the Methods section is a bit confusing. It starts out with a very brief mention of the data, then goes on to present the idea of constructing life expectancies based on individual mortality data and showing some results based on this approach in Table 1, then moves to a short literature discussion (mentioned in point 1 above), and then finally presents the methods used. A restructuring of this section would be helpful.

3) Following point 2), I suggest that the authors create a specific Data section explaining in a bit more detail about the 2010 Census and NCHS data for readers who are not quite familiar with these datasets. It was not clear to me whether the authors had access to information at the individual for both partners in a couple? Also, what is the source of the life expectancies from these data? Maybe it is stated in another part of the paper.

4) It is not clear to me what data are used to create Table 1. Moreover, in general, tables should be self-explanatory, without having to read the text in detail. I suggest making a note to Table 1 with instruction about how to read it. Moreover, in line 197: When speaking about "Our calculations, …", I presume that the authors are speaking about the calculations leading to Table 1?

5) Methodological contribution: In order to assess the significance of taking proper account of intra-couple correlation in health and mortality rather than just using individual data, would it be possible e.g. to generate a table like Table 1, but just based on the data for couples?

6) I found the last four lines of the Conclusion confusing, especially point c), as I thought that the point of the whole exercise in the paper is to show the importance of taking marital status into account when calculating life expectancies for couples.

Specific and minor comments

Lines 94-98 in the Introduction more or less repeat the text in lines 65-68.
